# Forest litter and shrubs act as an understory filter for the survival of *Quercus mongolica* seedlings in Mt. Kwan-ak, South Korea

**DOI:** 10.1038/s41598-019-40624-4

**Published:** 2019-03-12

**Authors:** Uhram Song

**Affiliations:** Department of Biology and Research Institute for Basic Sciences, Jeju National University, Jeju, 690-756 Korea

## Abstract

Forest succession from *Pinus* to *Quercus* has often been observed in temperate forest, although the succession mechanism is not clearly understood. This study investigated factors that affect the succession of forests from pine to oak, using forest vegetation inventory data at plots at Kwan-ak mountain in Korea. Analyses of understory canopy coverage, light intensity, and tree numbers and ages in *P. densiflora* forests indicate that *Q. mongolica* can only invade these forests before understory shrub establishment. The results from analyses of all environmental factors indicate that similar adverse effects from environmental factors occur in established *P. densiflora* and *Q. mongolica* forests that inhibit *Q. mongolica* seedling survival. However, the observed survival rate of *Q. mongolica* seedlings under *P. densiflora* during winter were much higher than *Q. mongolica* seedlings under *Q. mongolica* trees, and it is due to accumulated snow over *Q. mongolica* forest litter which breaks or inhibits the emergence of *Q. mongolica* seedlings. Protecting seedlings with plastic cups significantly increased the survival rate which confirms that forest floor litter acts as a filter for the regeneration and succession of *Q. mongolica* forests. This paper thus concludes that understory shrubs and forest litter affect the succession dynamics of *P. densiflora* and *Q. mongolica* forests.

## Introduction

Although understanding the concept of Forest succession is important in ecology^1^, yet the ecological mechanisms of this succession are still a subject of debate^1^ and has remained a challenging scientific problem for more than a century^2^. In temperate ecosystems, a pine stand is typically succeeded by hardwoods, unless interrupted by forest fires^3^. During the earlier stage of succession, pines tower over other woody species in temperate forests to form a canopy with an understory of herbaceous and hardwood tree species^4^. The community is eventually replaced by hardwood trees in the later stage. In East Asia, the predominant successional pattern has been observed as that from *P. densiflora* to *Q. mongolica*^5^.

Despite the ample observational evidence of the successional pattern, analyses have been lacking on factors that mediate such succession processes in many temperate ecosystems. Direct experimental research is often not feasible because of the longevity of the species in these communities, while the literature itself is divided between divergent hypotheses of different ecological succession theories^1^. In a general term, studies have suggested that natural forest succession can be triggered or affected by many endogenous and exogenous factors, including flooding, sedimentation^6^, large-scale and frequent natural disturbances^7,8^, effects of previous species on resource availability and resistance to invasion^8^, and interactions with other species^9^. On a finer scale, forest succession can be affected by altered soil conditions^10^, vegetation height, canopy cover^11^ and even by herbaceous understory plants^12^. Recent studies have pointed out complex effects of multiple factors including climate, nitrogen deposition, species composition, light and water availability and litter layer that affect succession^13^. Despite the accumulated research about the forest succession, however, factors influencing pine to oak succession dynamics have not been adequately addressed that each studies showed different hypothesis about the reason of such patterns^14^.

Despite the lack of research and the complexity in interactions, this study examines factors influencing pine to oak forest succession, drawing from a set of key factors that have been suggested to accelerate or inhibit succession in previous research. Namely, succession from *Pinus* to *Quercus* forest within our study area is believed to be affected by light intensity, canopy cover, differences of litter, and gap formation within the canopy, although no published local research provides direct evidence to support these beliefs. Also previous researches found which relates forest succession and litter are modeling based research^12^ or researches of other continent^15^ or about litter changing soil^16^ which needs more test for research area of this paper.

To draw upon and verify such beliefs, this study selects two factors that may affect the succession dynamics from *Pinus* to *Quercus* forests. First, the study analyzes whether one of the environmental factors including light intensity shows significant differences between *Pinus* and *Quercus* forests. Then it goes on to verify the effect of forest litters, which has been assumed to be the major factor that causes difference of seedling survival rate between *Pinus* and *Quercus* forests.

## Results

### Quadrat sampling and environmental factors

In each *P. densiflora* quadrat, there were an average of 19.8 *P. densiflora* trees, 7.3 *Q. mongolica* trees, and 20.8 *Rhododendron mucronulatum* shrubs (Table S1). In each *Q. mongolica* quadrat, there were an average of 9.5 *Q. mongolica* trees, 1.8 *P. densiflora* trees, and 5.5 *R. mucronulatum* shrubs (Table S1 in Supplementary Information). The average *P. densiflora* and *Q. mongolica* heights in *P. densiflora* and *Q. mongolica* quadrats were 6.3 and 10.6 m, respectively (Table S2 in Supplementary Information). The average *R. mucronulatum* height was approximately 1.7 m in all quadrats (Table S2). Table 1 presents the ages of trees in the quadrats. Both *Q. mongolica* and *P. densiflora* were approximately 30 years old (Table 1). The age of *Q. mongolica* in *P. densiflora* quadrats was approximately 10 years, whereas the age of *P. densiflora* in *Q. mongolica* quadrats was greater than 25 years (Table 1). The average age of *R. mucronulatum* in all quadrats was approximately 15 years (Table 1). The average Basal Area of *P. densiflora* in *P. densiflora* quadrats was 63.3 m^2^/ha and 89.1 m^2^/ha for *Q. mongolica* in *Q. mongolica* quadrats.

**Table 1:** Tree and shrub ages in *P. densiflora* and *Q. mongolica* forests.

	P. densiflora	Q. mongolica	R. mucronulatum
*P. densiflora* forest	30.8 ± 1.2	9.8 ± 0.4	16.0 ± 0.5
*Q. mongolica* forest	28.1 ± 1.2	25.8 ± 0.6	14.8 ± 0.6

Values represent mean ± SE of 12 replicates.

*Values of *P. densiflora* in *Q. mongolica* forest represent mean ± SE of seven replicates.

**Figure 1:**
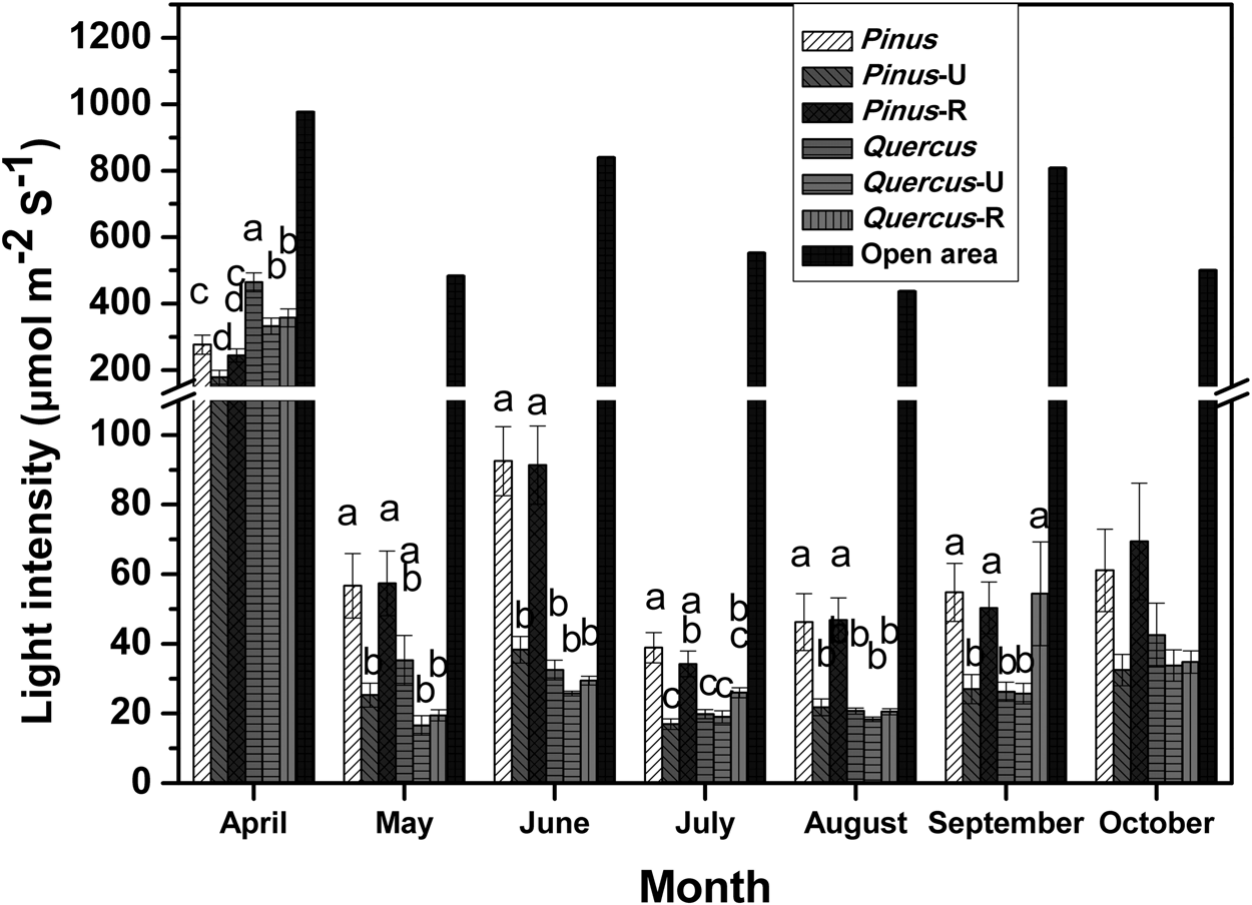
Monthly light intensity (mmol m^−2^ S^−1^) of *P. densiflora* and *Q. mongolica* forests. Bars and error bars represent mean ± SE of 24 replicates. Bars with the same letter are not significantly different at the *P* = 0.05 level. U, understory (measured at 10 cm height); R, removed (understory vegetation was removed).

The light intensity on sunny days in each quadrat at three points (above and below the understory vegetation and with the understory removed) is shown in Fig. 1.

In April, when *Q. mongolica* leaves have not yet emerged, the light intensity in *P. densiflora* forests was significantly lower than that in *Q. mongolica* forests. However, after early spring, the light intensity in *P. densiflora* forests was significantly higher than that in *Q. mongolica* forests (Fig. 1). The monthly total radiation measured by the film-type PPFD meter showed similar results (Table S3 in Supplementary Information). The light intensity (Fig. 1) and monthly radiation (Table S3) did not significantly differ for treatments below the understory (designated with ‘U’) between *Q. mongolica* and *P. densiflora* quadrats. The patterns of canopy coverage for dominant areas of both species were similar. Although canopy coverage at breast height was significantly higher for *Q. mongolica* forests (Table 2), canopy coverage below the understory vegetation did not significantly differ from that of *P. densiflora* forests.

**Table 2:** Canopy coverage (%) of *P. densiflora* and *Q. mongolica* forests in September 2006.

Forest	At breast height	Understory	Removed
*P. densiflora* forest	74.6 ± 1.5^b^	81.7 ± 0.9	75.1 ± 1.7^b^
*Q. mongolica* forest	84.2 ± 0.6^a^	81.5 ± 0.6	82.9 ± 0.5^a^

Values represent mean ± SE of 20 replicates.

Understory measured at 10 cm height; Removed, understory vegetation was removed.

There were no significant differences in total N, NH_4_^+^-N, and moisture contents of soil in *Q. mongolica* and *P. densiflora* quadrats (Table S4 in Supplementary Information) in spring. By contrast, the soil contents of NO_3_^−^-N, organic matter, and summer moisture were significantly higher in *Q. mongolica* forests than in *P. densiflora* forests. N contents of plant leaves were significantly different; *P. densiflora* leaves had significantly lower N contents than other species (Table S5 in Supplementary Information).

### Seedling survival rate

**Figure 2:**
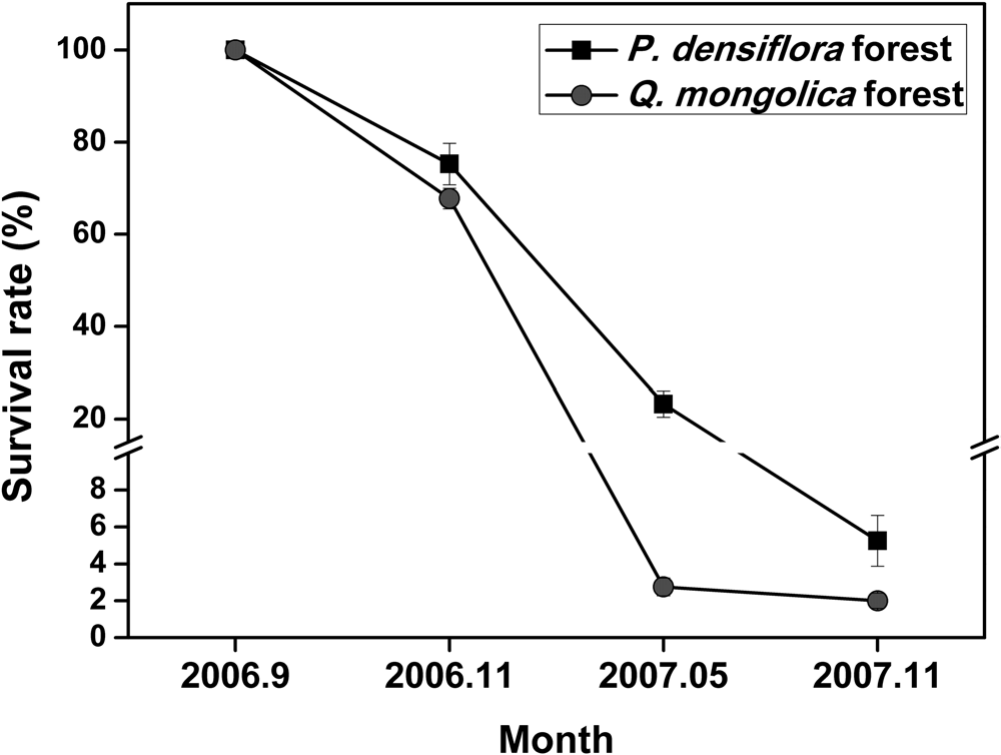
Survival rates of *Q. mongolica* seeds in *P. densiflora* and *Q. mongolica* forests. Symbols and error bars represent mean ± SE of four replicates. One hundred seeds were planted in each quadrat.

The survival rate of *Q. mongolica* seedlings in each quadrat rapidly declined after the winter season, and fewer than 5% of seedlings survived after the next year’s growing season (Fig. 2).

Table 3 presents the survival rate of naturally germinated *Q. mongolica* seedlings between 2007 and 2008. After the winter season, the seedling survival rate in *Q. mongolica* and *P. densiflora* forests were significantly different. In *Q. mongolica* forests, the seedling survival rate was less than 9% (Table 3).

**Table 3:** Numbers of *Q. mongolica* seedlings in 10 × 10 m plots in *P. densiflora* and *Q. mongolica* forests between 2007 and 2008.

	October (2007)	May (2008)	Survival rate (%)
*P. densiflora* forest	16.8 ± 2.2^b^	6.0 ± 0.9^b^	39.1 ± 10.0^a^
*Q. mongolica* forest	133.5 ± 22.4^a^	9.5 ± 1.4^a^	8.8 ± 3.4^b^

Values represent mean ± SE of four replicates.

Values in a column with the same letter are not significantly different at the *P* = 0.05 level.

Table 4 shows the number of seeds, germination rate, and seedling survival rate for *Q. mongolica* between 2008 and 2009. More than 95% of seeds germinated, but the *Q. mongolica* seedling survival rate through the next year was approximately 3% for *Q. mongolica* forests and more than 50% for *P. densiflora* forests (Table 4). However, the absolute numbers of *Q. mongolica* seeds and seedlings in *P. densiflora* forests were too small for statistical comparison with the survival rate in *Q. mongolica* forests.

**Table 4:** Numbers of *Q. mongolica* seeds and seedlings in a 1 × 1 m plot in *P. densiflora* and *Q. mongolica* forests between 2008 and 2009.

	Seeds (2008)	Seedlings (2008)	Seedlings (2009)	Survival rate (%)
*P. densiflora* forest	0.21 ± 0.08^b^	0.21 ± 0.08^b^	0.11 ± 0.06	50.0 ± 22.36
*Q. mongolica* forest	12.54 ± 1.46^a^	11.96 ± 1.36^a^	0.32 ± 0.12	3.11 ± 1.26

Values represent mean ± SE of 28 replicates (six replicates for ‘survival rate’ of *P. densiflora* forest).

Values in a column with the same letter are not significantly different at the *P* = 0.05 level.

Survival rate of *Q. mongolica* seedlings in *P. densiflora* forest was measured only in quadrats that originally had seedlings (6). Numbers of seeds were measured in September 2008 and numbers of seedlings were measured in October 2008 and May 2009.

### Litter weight affects seedling survival

The weight of litter on the ground significantly differed between *Q. mongolica* and *P. densiflora* forests (Table 5). Especially after heavy snow, the weight of litter and accumulated snow was greater than 7 kg m^−2^. Without snow, the weight of *Q. mongolica* litter with moisture was greater than 1 kg m^−2^. The seedling emergence rate was significantly lower for seeds germinated in pots covered with *Q. mongolica* litter at similar weight per unit area as that observed in *Q. mongolica* forests compared with those covered with *P. densiflora* litter (Table S6 in Supplementary Information). Seedlings protected from the weight of litter and accumulated snow by a physical barrier (cup) had significantly higher survival rate compared with control seedlings (Table 6). In *Q. mongolica* forests, the survival rate of protected seedlings was approximately 10 times higher than that for unprotected seedlings.

**Table 5:** Weight of litter (g) in *P. densiflora* and *Q. mongolica* forests.

	Litter trap	Litter on ground	Litter with snow	Litter without snow
*P. densiflora* forest	169 ± 61	256 ± 13^b^	7133 ± 114	318 ± 14^b^
*Q. mongolica* forest	134 ± 12	644 ± 37^a^	7725 ± 237	1175 ± 98^a^

Values represent mean ± SE of 12 replicates.

Values in a column with the same letter are not significantly different at the *P* = 0.05 level.

Litter trap and Litter on ground are litter dry weight; Litter with snow and Litter without snow are litter fresh weights. Litter trap and Litter on ground were measured in March 2008. Litter with snow was measured after 11 mm of precipitation (snow) in January; Litter without snow was measured 2 weeks later after snow melt.

**Table 6:** Survival rate (%) of *Q. mongolica* seeds and seedlings between 2008 and 2009.

	Seedlings with cup (2009)	Seedlings without cup (2009)
*P. densiflora* forest	67.5 ± 2.5^b^	48.8 ± 2.3^a^
*Q. mongolica* forest	77.5 ± 1.6^a^	5.0 ± 1.9^b^

Values represent mean ± SE of eight replicates (each replicate represents survival rate of ten seedlings).

Values in a column with the same letter are not significantly different at the *P* = 0.05 level.

Seedlings were selected in October 2008 and seedling survival rates were measured in May 2009. Cups were placed in October 2008.

## Discussion

The number of individuals and the age of each species in different forest stands indicate the successional dynamics of the studied stands and the influential factors that potentially determined the dynamics. *P. densiflora* forests had approximately twice the number of *P. densiflora* (average 19.8 trees) and approximately four times the number of *R. mucronulatum* shrubs (average 20.8) in each quadrat compared with *Q. mongolica* forests (average 9.5 individuals of *Q. mongolica* and average 5.5 individuals of *R. mucronulatum* shrubs; Table S1 in Supplementary Information). The *P. densiflora* quadrats had an average of 7.3 *Q. mongolica* trees, and the age of *Q. mongolica* trees in *P. densiflora* forests (average 9.8 years) was relatively young. This suggests that *Q. mongolica* trees appeared after the formation of the *P. densiflora* forest (Table 1) and that *Q. mongolica* began succession into the established *P. densiflora* forest. By contrast, the ages of *P. densiflora* (average 28.1 years) and *Q. mongolica* (average 25.8 years) trees were similar in *Q. mongolica* forests, indicating that *P. densiflora* and *Q. mongolica* trees established at similar times (Table 1). The numbers and ages (average age approximately 15 years) of *R. mucronulatum* shrubs (Table S1 and Table 1) indicate that this species is introduced later in both *Q. mongolica* and *P. densiflora* forests, and only becomes successfully established as the dominant understory vegetation in *P. densiflora* forests (Table S1 in Supplementary Information).

The mean heights of all individuals of *P. densiflora* and *Q. mongolica* in *P. densiflora* and *Q. mongolica* quadrats were 6.3 and 10.6 m, respectively (Table S2 in Supplementary Information). The mean heights of trees forming the canopy structure were 7.1 ± 0.2 m (mean ± SE of 58 replicates) for *P. densiflora* and 11.2 ± 0.2 m (mean ± SE of 33 replicates) for *Q. mongolica*. The mean height of *R. mucronulatum* in all quadrats was approximately 1.7 m (Table S2 in Supplementary Information).

Despite the difference in canopy heights, light intensity did not appear to be the major factor to influence succession, nor did the canopy coverage. Figure 1 shows the light intensity during sunny days at three points (above and below the understory vegetation and with the understory removed). In April, when *Q. mongolica* leaves have not emerged, the light intensity of *P. densiflora* forest was significantly lower than that of *Q. mongolica* forest because only *P. densiflora* trees had foliage. However, after early spring when the leaves of other species had emerged, the light intensity of *P. densiflora* forest was significantly higher than that of *Q. mongolica* forest (Fig. 1) when observed above the understory vegetation. However, the the light intensity of *P. densiflora* forest under the *R. mucronulatum* was no different than that of *Q. mongolica* forest. The monthly total radiation measured by film-type PPFD meter showed similar results (Table S4 in Supplementary Information). The light intensity (Fig. 1) and monthly radiation (Table S4) did not significantly differ between *Q. mongolica* and *P. densiflora* quadrats for below understory treatments (designated with ‘U’). The canopy coverage of both areas of dominant species showed similar patterns. The canopy coverage at breast height (above understory vegetation) was significantly higher for *Q. mongolica* forest (Table 2), although the canopy coverage below the understory vegetation did not significantly differ. This important result indicates that light intensity and canopy coverage are not the major factors controlling differences in succession between *Q. mongolica* and *P. densiflora* forests because late-successional species would receive similar light intensity in both forests.

The results are at odds with previous literature of same study site that suggested that the canopy coverage and the light intensity are believed to be the main factors determining succession from *Pinus* to *Quercus* forest^5^. The same study also lists the canopy formation as one of such main factors^5^, but this study’s findings also suggest otherwise. Our results indicate that even when a canopy gap is introduced by uprooting, *Q. mongolica* seedlings in *P. densiflora* forests would still receive less light because the understory canopy of *R. mucronulatum* is high. Similar results were reported in other *Pinus* forests; understory light intensity was not substantially higher than that of broad-leaved forest because of the understory vegetation^17^ and the canopy’s effects on understory patterns^18^. This is a similar effect as some understory plants work as an understory filter, although most studies of understory filters focused on herbaceous species^19,20^. Dense understory layers working as understory filters have been reported worldwide in forests that alternate between regeneration and succession^21^. Some early successional species cannot invade and grow in the presence of adults of their own or other species^8^. Similar effects may occur in other *Pinus* forests with understory vegetation, and not only in the study site of the current research. *Quercus mongolica* seedlings struggling under the canopy coverage change would not benefit from an occasional canopy gap as they still receive reduced amount of light due to the understory vegetation of *P. densiflora* forests. From this, we can conclude that the shrubs function as an understory filter that blocks succession of *P. densiflora* forests. With the understory filter, *P. densiflora* forests do not provide better incident light conditions than *Q. mongolica* forests.

Soil analyses indicated some differences between the two forest stands but did not further clarify the factors behind an observed difference in seedling survival rates. The soil contents of NO_3_^−^-N, organic matter, and summer moisture were significantly higher in the *Q. mongolica* forest. By contrast, soil contents of total N, NH_4_^+^-N, and moisture did not significantly differ between *Q. mongolica* and *P. densiflora* quadrats (Table S4 in Supplementary Information). Spring is usually the dry season at the study site and showed low soil moisture contents that plants are subjected to drought stress^22^. Soil nitrogen contents did not substantially differ, so soil conditions would not be a factor affecting the survival of seedlings. Leaf N contents did significantly differ; *P. densiflora* leaves had significantly lower N contents than other species (Table S5 in Supplementary Information). As the environmental factors were similar such as the overstory canopy coverage, light intensity, and soil conditions between the two forests, factors affecting the *Q. mongolica* seedling survival and the succession from *Pinus* to *Quercus* forest remained unclear. The combined results suggest that there could be other factors that render *P. densiflora* forests more beneficial than *Q. mongolica* forests for *Q. mongolica* seedlings.

Actual differences in the seedling survival rate between *Q. mongolica* and *P. densiflora* forests could predict future succession dynamics. The *Q. mongolica* seedling survival rate in each quadrat rapidly declined after the 2006−2007 winter season, and less than 5% of seedlings survived after the growing season (Fig. 2). The differences between species became more apparent during the 2007−2008 experiments (Table 3). After the winter season, the seedling survival rates in *Q. mongolica* and *P. densiflora* forests were 9% and approximately 40% (Table 3). *Quercus mongolica* had abundant acorn production in 2008, and more than 1,200 acorns were produced in each 10 × 10 m *Q. mongolica* quadrat. Table 4 shows the number of seeds, the germination rate, and the seedling survival rate of *Q. mongolica* in 2008−2009. More than 95% of *Q. mongolica* seeds germinated, but only approximately 3% of seedlings survived in *Q. mongolica* forests, whereas greater than 50% survived in *P. densiflora* forests (Table 4), although the absolute numbers of seeds and seedlings in *P. densiflora* forests was too small for statistical significance. The observed low survival rate of *Q. mongolica* seedlings in *Q. mongolica* forests may explain the successional dynamics from *Pinus* forest to *Quercus* forest. Unlike the *Q. mongolica* seedlings, the numbers of *P. densiflora* seedlings found in total 28 quadrats were only 2 in *Q. mongolica* forests and only 6 even in *P. densiflora* forests. The numbers of *P. densiflora* seedlings were too small for any test and implies that the *P. densiflora* forest will likely not persist due to such poor regeneration. The numbers and ages of trees (Table S1 and Table 1) indicate that *P. densiflora* forests have more surviving *Q. mongolica* seedlings than *Q. mongolica* forests, even though the absolute number of seeds and seedlings is much smaller than in *Q. mongolica* forests (Tables 3 and 4).

The factors were not immediately clear from the initial set of results that affect *Q. mongolica* the difference in seedling survival rates in *Q. mongolica* and *P. densiflora* forests. However, careful field observations identified many *Quercus* seedlings that were broken and other seedlings that remained buried in the forest floor litter, which may have affected the general rate of seedling establishment. A second set of analyses, thus, were performed to investigate the effects of litter on seedling mergence and survival.

The weight of litter on the ground significantly differed between *Q. mongolica* and *P. densiflora* forests (Table 5). The *Pinus densiflora* litter contained broken branches and shed leaves. When the branches were excluded, the average weight of the *P. densiflora* litter trap was 67 ± 21 g·m^−2^. After heavy snow, the weight of *Q. mongolica* litter and accumulated snow was more than 7,700 g·m^−2^. Even without snow, the weight of *Q. mongolica* litter with moisture was greater than 1,000 g·m^−2^. This indicates that, although seedlings can easily emerge through the litter of pine needles and snow in *P. densiflora* forest, oak litter completely blocks the seedling emergence and the seedlings receive the much heavier weight of combined litter and snow in the *Q. mongolica* forest. *Quercus mongolica* seeds (acorns) germinate at the litter surface, rapidly establish a root that extends downward, and the acorns become exposed on top of or above the litter. This makes them susceptible to breakage due to the weight and pressure of shifting litter and snow cover. Even the seedlings that become well attached to the ground and are not broken can still be inhibited from a complete emergence due to accumulated layers of litter and snow cover. This is consistent with this study’s field observations where we saw many *Q. mongolica* seedlings in the spring that survived the winter but could not emerge out of the litter, which is a sort of mechanical inhibition that affects forest dynamics and succession^21^.

To study the emergence rate, further analyses were performed on the effects of wet litter on seedling emergence. Seedling emergence rates in pot experiments were significantly reduced by *Q. mongolica* litter supplied at a similar weight per unit area as that observed in the quadrats (Table S6 in Supplementary Information). *Pinus densiflora* seedlings were able to emerge through *Q. mongolica* litters, but some *Q. mongolica* seedlings were not able to emerge after germination. Approximately 50% of *Q. mongolica* seeds were able to germinate and emerge through *Q. mongolica* litter. The edges of the pots had greater seedling emergence due to reduced litter coverage, whereas under field conditions the litter forms a continuous layer that suppresses seedling emergence more uniformly.

To test the actual effects of litter under field conditions, physical barriers were generated by surrounding seedlings with open-top cups. These barriers protected seedlings from the weight and/or pressure of shifting forest litter and the accumulating snow. The protected seedlings had significantly improved survival rates compared with those of controls (Table 6). The survival rate of *Q. mongolica* seedlings in *Q. mongolica* forest was approximately 10 times higher (49%) than controls (5%) when protected by the physical barrier. This result indicates that oak seedlings are negatively affected by the oak litter, either due to breakage or due to blocked emergence in the spring. This experiment also showed that *P. densiflora* litter does not adversely affect *Q. mongolica* seedlings as much as *Q. mongolica* litter because the seedling have similar survival rates with and without the physical barriers (Table 6). This result explains why *Q. mongolica* seedlings showed better survival in *P. densiflora* forests (Table S1). This is a new finding as, although effects of forest litters on seedling survival rate have been previously reported, but in most cases the effects were about the changed light intensity, the production of chemical compounds and acidification^13^. However, this paper suggests new aspects of forest litter’s influence that directly and negatively affects survival of seedlings in combination of snow and moisture (litter weight).

This section considers a few factors that may influence the study results or the representativeness of the study results outside the design as follows. It could be possible that the cups might protect acorns from freezing; however, acorns under *Q. mongolica* litter with accumulated snow were also insulated, and as *Q. mongolica* seedlings in *P. densiflora* litter with less insulation (low temperature) shows a high survival rate, we can rule out the temperature’s effect on the seedling survival. Another argument can suggest that seedlings surrounded by cups also might be protected from animal feeding during the winter, which would increase seedling survival independent of the litter weight effect. Indeed, low acorn germination rates due to animal feeding have been reported in Korean mixed-oak forests^23^. However, in the study area, approximately 50% of the acorns and seedlings survive in *P. densiflora* forest, suggesting that animal feeding would not substantially affect the total survival rate of *Q. mongolica* acorns and seedlings in our research area. Also, an unstudied portion of seedlings can make up the total population in the field site. The author tried to minimize litter disturbance while searching for acorns and seedlings, and there could be acorns and seedlings that became well established underneath several layers of litter that were not observed in the field studies. These buried acorns and seedlings, however, are not anticipated to represent the majority of cases. The results of the survival experiments represent typical seedlings that emerge above the litter, which are a small fraction of the population. One last possibility should be considered: Rapid changes in the environment would reduce *Q. mongolica* seedling survival in the field. A previous study of the same research area (Kwan-ak Mountain, Seoul, Korea) suggested that rapid environmental changes between 1972 and 2010, including soil acidification and the decline in mineral ions such as potassium (reduced from 0.67 to 0.25 cmol kg^−1^) and calcium (reduced from 3.20 to 0.87 cmol kg^−1^), would affect the survival of plants in the study area^5^. This could be due to the urbanization of Seoul that could affect plants in the study area. The expected effect from acidification or mineral content, however, could be inconsequential during the winter, the study period for seedling survival, as physiological activity is low during the season. Although further studies investigating the effects of environmental changes on oak seedling survival should be performed, the environmental changes are not related to the effect of the litter weight.

For further studies, it should be noted that the author had planned a further study of other *Q. mongolica* forests to verify effects of litters on survival of seedlings. Other sides (north-western slope) of Kwan-ak mountain (2009 and 2010) or other mountains in Gyeonggi province (2011 and 2014) of Korea were planned for additional research. However, acorn production was very low during 2009−2010, and since acorns are used for food ingredients in local area, almost all acorns were collected by humans even before measuring numbers of fallen acorns (year 2011 and 2014). Therefore, additional data collection in the same study area has not been successful. Another idea for further research is to expand the geographic scope to see whether the site has similar characteristics as other temperate forests in East Asian countries such as China and Japan. As both countries’ territories span across a wider range of latitudes, the countries have a wider variety of forest types. Despite the wider variety, many research report forests where *Pinus* and *Quercus* species coexist in China^24,25^ and Japan^26^, indicating similar dynamics may happen. Also, some papers have made a similar prediction of an increase in *Quercus* species during forest succession in China^27^ or a similar succession pattern from *Pinus* to *Quercus* species in Japan^28,29^ showing the case of the study site is applicable. Such similarities indicate that this paper’s study of Kwan-ak Mountain can inform further studies on forest dynamics consisting of *Pinus* and *Quercus* species, especially those of East Asia, where there is more similarities in species composition and climate. Although there should be more caution in applying the findings on the roles of forest litters and understory shrubs to other *Pinus-Quercus* forests around the world since the major species are very different and there will be very different snowfall, it can still inform further studies as one of the potential factors that can include the dynamics. Thus, this hypothesis should be tested in other sites where succession from *Pinus* forest to *Quercus* forests are observed. As plants of the same genus may have many similar features, the results may show some similarities.

In conclusion, the results of this study show that the litter in *Q. mongolica* forests and the presence of shrubs in *P. densiflora* forests affect the *Q. mongolica* seedling survival, and ultimately affect the succession of *Pinus-Quercus* forests. Traditionally, it was believed that the higher light intensity and the reduced overstory canopy coverage in *Pinus* forest accounted for the *Quercus* succession. However, understory shrubs in *Pinus* forest reduced the light intensity and increased understory canopy coverage, mitigating the light-increasing impact of the overstory canopy of *Pinus* forest. *Pinus* forest was indeed brighter than *Quercus* forest, but the incident light enabled understory shrubs to become established, the shrubs reduce light intensity in the understory where *Q. mongolica* seedlings become established during succession. The average age of *Q. mongolica* trees was 9.8 years, indicating that the oak trees become established only a few years after establishment of the understory shrub (*R. mucronulatum* average age: 16.0 years old). *Quercus mongolica* was not able to generate more young trees for succession (Table 1) due to understory filtering by shrubs and other environmental factors, indicating that *Pinus* forest conditions are not significantly better than those of *Quercus* forest. Establishment of *Q. mongolica* individuals before the shrub establishment would facilitate succession. The results of our studies on litter provide insights into why *Pinus* forest is preferable for *Q. mongolica* seedling survival and eventual succession. The accumulated litter and snow of *Q. mongolica* forest adversely affects *Q. mongolica* seedling survival and emergence.

During the initial stage of *Q. mongolica* introduction, *Q. mongolica* litter would have benefits such as the moisture-holding capacity and insulating properties during the winter. After the *Q. mongolica* establishment, litter would have adverse effects that block regeneration. In Korea, *Quercus acutissima* drops litter in the spring rather than in the fall. This trait may have been developed to prevent pressure on the seedlings during the winter. However, *Q. acutissima* usually inhabits lower elevations with less snow accumulation^30^. Further research on spring foliage strategies should be investigated. Several complex factors should be noted: First, acorns are harvested by humans as a food resource in Korea, which complicates experimental analyses on the seedling survival, which may happen in other sites. Secondly, Korea is currently experiencing dramatic effects of climate change due to global warming^31^, which could be anticipated to reduce snow accumulation in the future, which should affect seedling survival during winter. Further analysis of *Pinus-Quercus* succession in other countries would help to establish relationships between the global climate change and the seedling survival of *Quercus* species.

## Methods

### Species selection and site description

Korean red pine (*Pinus densiflora* S. et Z.) is one of the most abundant plant species in Korea^32^. Oak species also are abundant in Korea. Mongolian oak (*Quercus mongolica* Fisch. ex Ledeb.) is a representative oak species in this region^33^. *Pinus densiflora* forests often are invaded by *Q. mongolica* as a pattern of succession^34^.

The study area is the northeastern slope of Kwan-ak Mountain, Seoul, Korea. The forest is a temperate forest, which is believed to undergo succession from pine forest to oak forest, and eventually to broadleaf forest populated by trees such as *Carpinus*^35,36^. The study site shows typical vegetation pattern of temperate forests that dominated by *P. densiflora-Q. mongolica* or *Q. serrata* community^5,33,34,37^, that could represent temperate forests in Far east Asia. *Pinus densiflora* forests are being invaded by progressive succession of *Q. serrata* and *Q. mongolica* in the shrub or sub-tree layer^34^.

The slope is dominated primarily by *P. densiflora* or *Q. mongolica*; each species dominates in many patchy areas along the slope. The central geographic coordinates of the study area are 126° 58′ 11.28″ N and 37° 26′ 43.98″ E, with 470 m elevation above sea level.

### Experimental design

#### Quadrat sampling

In 2006, four 10 × 10 m quadrats were established in which one of the species was dominant, for a total of eight quadrats. Each quadrat was at least 20 m distant from any other quadrat. The numbers of species and individuals within each quadrat were recorded. Individual ages were measured in 2009 by counting the annual rings from core samples of three individual trees that were randomly chosen. Tree height was measured using Haglöf vertex IV (Haglöf Sweden AB, Långsele, Sweden). *Rhododendron mucronulatum* was the most dominant understory vegetation species; the understory coverage in each quadrat also was measured at this time. Within each 10 × 10 m quadrat, 2 × 2 m quadrats were established, and shrubs inside the small quadrats were cut to compare the effects of shrub species on canopy coverage, light intensity, and photon flux density. These small quadrats were used to conduct the ‘understory vegetation removed’ treatment. In April of 2006, 4 subsamples in each quadrats were gathered as representative soil sample for each quadrats.

#### Monitoring the survival rate of Q. mongolica seedlings

In 2006, 100 germinated seeds of *Q. mongolica* were planted in the 2 × 2 m areas within the 10 × 10 m quadrats. Seedling survival rates were monitored for 1.5 years. After the newly matured seeds were shed from *Q. mongolica* in September 2007, the numbers of germinated seeds and seedlings were measured. The seedling numbers were measured again in late spring 2008, and the seedling survival rates in *Q. mongolica* and *P. densiflora* quadrats were compared. After seeds were shed in 2008, the numbers of seeds, seeds germinated after 1 month, and surviving seedlings in the late spring of 2009 were measured in seven 1 × 1 m quadrats, which were arranged along an imaginary diagonal line starting at the left top edge and proceeding toward the right bottom edge, giving a total of 28 quadrats for each species.

#### Litter weight determination

In September 2007, a 0.5 m^2^ litter trap (1 m above ground) and a 0.2 m^2^ litter trap (at the forest floor level) were established with three replicates for each quadrat. The trapped litter was harvested in March 2008. This measured litter represents the total amount of fallen litter (1 m above ground) or accumulated litter (at the forest floor level) that occurs from the time of seed shedding until the next spring. The litter weights of the 0.5 m^2^ traps including accumulated snow were measured in January 2008 with three replicates for each quadrat. There was a snow event with a reported 11 mm of precipitation just before litter collection^38^. The exact amount of precipitation at the site was not available during the field research, but approximately 9 cm of accumulated snow was observed at the site. The snow melted 2 weeks later and there was no reported precipitation for that same period^38^; the litter weights were measured again at that time to determine the weight without snow. Every litter weight measurement was used to calculate the litter weight in one square meter.

#### Determining the emergence rate of Q. mongolica seedlings

To test whether litter inhibits seed germination or seedling emergence, seeds of *Q. mongolica* or *P. densiflora* were planted in pots (20 cm diameter and 20 cm height) in April 2008. Five seeds were planted in each pot, and litter collected from *Q. mongolica* or *P. densiflora* forests were layered on top of the soil at the same rate and weight as that calculated from the measured litter in the 0.2 m^2^ litter trap placed at the forest floor, which created a similar environment as the actual field environment. The measured litter weights at the floors of the *Q. mongolica* and *P. densiflora* forests was 640 and 256 g m^−2^, respectively. Therefore, the pots with *Q. mongolica* and *P. densiflora* seeds were covered with 20 and 8 g of litter (dry weight), respectively. The pots were placed in the *Q. mongolica* forest understory, and germination and emergence rates were measured in August 2008.

#### Protection of seedlings with a physical barrier

In October 2008, 20 fresh *Q. mongolica* seedlings were selected; ten seedlings received physical protection and ten seedlings received no protection. The protection was provided by a transparent plastic cup (9 cm upper diameter and 6 cm lower diameter) with the bottom of the cup removed. The cup was placed upside-down over the seedling into the soil and fixed to the ground with long nails, which formed a small open-top chamber surrounding the seedling. This physical barrier protected seedlings from the covering litter and accumulated snow. Two sets of treatments (with and without cups) were established in every quadrat. The seedling survival rates with and without the cup were measured in May 2009.

### Analytical methods

#### Determining canopy coverage, light intensity, and photosynthetic rate

Five random points of each quadrat were chosen and pipes (1.6 m height) were installed. Photographs of the canopy cover and light intensity were taken from the top of the pipe (1.6 m height). A film-type photosynthetic photon flux density (PPFD) meter was placed at the top of the pipe to measure PPFD. Five random points were selected to photograph canopy cover and measure light intensity in the understory vegetation at a measured distance of 10 cm above the ground. Measuring canopy cover and light intensity at both 1.6 m and 10 cm enables us to differentiate the effects of canopy cover (overstory vegetation) and understory vegetation.

Canopy photographs were captured using a semi-fisheye lens (HD-3031PRO, Raynox, Japan) and a digital camera. The digital images were processed and analyzed to estimate canopy cover using the ImageJ program (National Institutes of Health, USA)^39^. Light intensity was measured using a line quantum sensor (LI-191, LI-COR, Lincoln, NE) on a sunny day. The monthly total radiation was measured using a film-type PPFD meter (T-METER THS-470, Eko Instruments, Tokyo, Japan), which measures the integrated radiation based on the degree of fading of the sensor film. Films were replaced and analyzed every 10 to 20 days depending on the number of sunny days during the experiment.

#### Litter and soil analyses

Carbon and nitrogen contents of litter and soil were measured using an element analyzer (Flash EA 1112; Thermo Electron Co., USA). The total N, NH_4_^+^-N, and NO_3_^−^-N contents of soil were analyzed using a Kjeldahl protein/nitrogen analyzer (Kjeltec Auto 1035 System; Tecator AB, Denmark). Soil moisture content was measured by comparing weights before and after drying at 105 °C for 47 hours.

### Statistical analysis

Differences between two groups were evaluated using Student’s *t*-test for normally distributed variables or the Mann-Whitney U-test when normality assumptions were violated (SAS v. 9.1, SAS Institute Inc., USA). To compare multiple groups with normally distributed variables, data were analyzed by one-way PROC ANOVA. When a significant treatment effect was detected, post-hoc comparisons of the means were performed with Duncan’s multiple range test (SAS v. 9.1, SAS Institute Inc., USA). To compare multiple independent groups with non-normally distributed variables, data were analyzed using the Kruskal-Wallis test. Statistical significance was inferred when *P* < 0.05. Data are presented as mean ± standard error (SE).

## Supplementary information

supplementary information

## Data Availability

All data generated or analyzed during this study are included in this published article and its Supplementary Information file.
