# Supplementary material for: Forest litter and shrubs act as an understory filter for the survival of Quercus mongolica seedlings in Mt. Kwan-ak, South Korea
Source: Sci Rep. 2019 Mar 12;9:4193. doi: 10.1038/s41598-019-40624-4 (PMC6414525; doi:10.1038/s41598-019-40624-4)
Supplement: Supplementary file 1 — supplementary information [file 41598_2019_40624_MOESM1_ESM.pdf]

Forest litter and shrubs act as an understory filter for the survival of *Quercus mongolica* seedlings in Mt. Kwan-ak, South Korea

Uhram Song

Table S1. Numbers of trees per research plot (10m x 10m) in *P. densiflora* and *Q. mongolica* forest

	P. densiflora	*P. densiflora* Seedling	*Q. mongolica*	*Q. mongolica* Seedling	*R. mucronulatum*	Others
P. densiflora forest	19.8±1.5	1.5±0.6	7.3±2.3	6.8±1.5	20.8±3.7	5.0±1.1
Q. mongolica forest	1.8±0.5	0.0±0.0	9.5±0.6	3.8±0.6	5.5±1.6	5.5±0.9

Values represent mean ± SE of 4 replicates.

Table S2. Tree heights of *P. densiflora* and *Q. mongolica* forest

	P. densiflora	*Q. mongolica*	*R. mucronulatum*	Others
P. densiflora forest	6.3±0.2 (79)	4.8±0.3 (29)	1.6±0.3 (83)	4.6±1.1 (20)
Q. mongolica forest	6.1±0.3 (7)	10.6±0.4 (38)	1.9±0.3 (22)	7.4±2.2 (22)

Values represent mean ± SE.

* Values in parenthesis represents numbers of replicates.

Table S3. Monthly total radiation of *P. densiflora* and *Q. mongolica* forest (MJ/m^2).

	March	April	May	June	July	August	September	October
Pinus	167.6[b] (1.8)[#]	183.7[c] (2.2)	205.3 (3.9)	126.1[ab] (2.9)	179.8[a] (5.2)[##]	278.3[a] (5.5)	146.0[a] (2.6)	104.7[a] (2.3)
Pinus-U	158.4[b] (1.5)	179.0[cd] (2.4)	195.9 (1.6)[##]	115.4[c] (1.2)[#]	161.2[b] (3.1)[##]	263.9[abc] (4.4)	135.7[b] (0.9)[#]	96.4[b] (1.4)[##]
Pinus-R	162.4[b] (1.6)[#]	173.9[d] (1.4)[#]	203.7 (2.5)	129.5[a] (3.3)	180.4[a] (3.9)[#]	275.2[ab] (4.8)	141.0[ab] (1.8)	101.6[ab] (1.8)
Quercus	138.2[c] (3.8)	212.2[a] (2.1)	200.8 (1.7)	116.9[c] (1.1)[#]	174.0[ab] (4.6)[##]	261.1[abc] (4.6)[#]	134.9[b] (0.9)	99.4[ab] (0.7)
Quercus-U	186.0[a] (3.0)[#]	202.0[b] (2.4)	198.0 (1.3)	119.8[bc] (1.4)[#]	162.3[b] (3.5)[##]	251.5[c] (1.9)	134.8[b] (1.3)[#]	97.5[b] (1.0)
Quercus-R	187.2[a] (3.1)[#]	205.8[ab] (1.7)	200.7 (3.7)[##]	115.9[c] (1.3)	176.0[ab] (4.5)[#]	257.8[bc] (3.8)	137.3[b] (1.0)	97.0[b] (0.8)
Open area	520.5 (2.8)	545.0 (3.2)	527.0 (11.6)	826.1 (0.6)	297.6 (6.4)	821.0 (0.8)	526.4 (1.8)	428.5 (1.8)

Values represent mean (SE) of 16 replicates (3 replicates for Open area)

Values having the same letter are not significantly different at the 0.05 level.

*U: Understory (measured at 10 cm height)

*R: Removed (Understory vegetation is removed)

Values with '[#]' indicates one sample is missing during the research.

Values with '[##]' indicates two samples were missing during the research.

The survey periods of each months are, 13 days for March, 12 days for April, 10 days for May, 18 days for June, 13 days for July, 10 days for August, 15 days for September and 21 days for October (Values are recalculated as 30 days).

Table S4. Soil properties of *P. densiflora* and *Q. mongolica* forest

	T-N (%)	NH_4^+-N (mg/kg)	NO_3^--N (mg/kg)	Organic matter	Moisture (April)	Moisture (August)
P. densiflora	0.67±0.03	40.69±3.28	1.17±0.48	6.4±0.2	15.3±0.2	19.2±0.5
Q. mongolica	0.75±0.03	37.57±6.78	4.10±0.78	11.9±0.3	15.4±0.2	27.6±0.8
P value			< 0.001	< 0.001		0.0209

Values represent mean ± SE of 4 replicates.

Values in a column having the same letter are not significantly different at the 0.05 level.

*Only p-values less than 0.05 (significantly different) are shown.

Table S5. Carbon and nitrogen contents (%) of *P. densiflora* and *Q. mongolica* litter

	C	N
P. densiflora	45.22±0.19[a]	1.43±0.12[b]
Q. mongolica	45.20±0.20[a]	2.21±0.05[a]
R. mucronulatum	41.70±0.52[b]	2.12±0.01[a]

Values represent mean ± SE of 4 replicates.

Values in a column having the same letter are not significantly different at the 0.05 level.

Table S6. Seedling emergence rate of *P. densiflora* and *Q. mongolica* under litter treatments.

Species	*P. densiflora*		*Q. mongolica*	
Treatment	*P* litter	*Q* litter	*P* litter	*Q* litter
Emergence rate	78.0±7.6 (0.0±0.0)	48.0±6.8 (22.0±5.5)	76.0±5.0 (2.0±2.0)	52.0±5.3 (28.0±4.4)
P value	0.0129		0.0065	

Values represent mean ± SE of 10 replicates.

Values in a row having the same letter are not significantly different at the 0.05 level.

* *P* litter: covered with *P. densiflora* litter, *Q* litter: covered with *Q. mongolica* litter.

* Values within parentheses indicates germinated seeds but not emerged through litters.

Table S7. Recorded temperatures (°C) by treatments during winter season.

	Average	Daily lowest temperature	Lowest temperature recorded	Daily highest temperature	Highest temperature recorded
Inside the cup	-0.65	-2.62	-7.23	1.67	8.46
Without the cup	0.10	-1.04	-3.99	1.57	8.13

*Values represent mean of 3 replicates.

*Temperatures are measured from 28th of December, 2016 to 19th of February, 2017.

*Temperatures are measured every hour by HOBO UX-100 data logger.
